# The Role of the Pupil, Corneal Reflex, and Iris in Determining the Perceived Direction of Gaze

**DOI:** 10.1177/2041669518765852

**Published:** 2018-08-21

**Authors:** Stuart Anstis

**Affiliations:** Department of Psychology, University of California, San Diego, La Jolla, CA, USA

**Keywords:** iris, pupil, gaze, induced movement, eye movements, local motion

## Abstract

In specially constructed movies depicting moving eyes, the pupils, irises, and corneal reflexes moved independently and sometimes in opposite directions. We found that the moving pupils or the corneal reflex, not the moving irises, determined the perceived direction of gaze (online Movie 1). When the pupils and irises moved in opposite directions, the one with the higher Michelson contrast determined the perceived direction of gaze (online Movie 2).

It is of social value to be able to judge the direction of another person’s gaze; a common everyday question is “Is that person looking at me?” And we are good at making such judgments (Gamer & Hecht, 2007; Kobayashi & Kohshima, 1997), although systematic errors can occur (Anstis, Mayhew, & Morley, 1969; George & Conty, 2008; Gibson & Pick, 1963). We wondered what visible parts of another person’s eye are used in judging where they are looking. These visible parts include the white sclera, the colored iris, and the black hole of the pupil. The small white corneal reflex is not a part of the eye but is a reduced reflection of the illumination.

In all previous publications on the judgments of eye movements, the pupil and the iris have moved in lockstep (as in real life), with a stationary corneal reflex. But our artificial movies teased apart the components of the moving eye that drove judgments of eye direction, by uncoupling the pupil, iris, and corneal reflex and allowing them to move independently in different directions, as never happens in real life. (The movements were kept small enough that the pupil remained within the iris.) Online Movie 1 shows six stimulus conditions. Fifteen naive observers were asked in which direction the eyes were moving in each condition, as the eyes swung back and forth horizontally. To facilitate reports, two small target letters oscillated back and forth in opposite directions above the eyes. Observers simply reported their percepts in the six conditions by writing down a string of six letters. For the observers, all target letters were the same size, but in online Movie 1, the winning letters have been enlarged. Labels, not visible to the observers, have also been added to each condition. Table 1 shows the results.

**Table 1.** Movements of pupil, iris and reflex drive perceived directions of eye movements.

No.	Pupil	Iris	Reflex	Perceived direction	Remarks	%
1	←	←	x	←** A**	Control	100
2	←	x	x	←** D**	Pupils move and win	87
3	X	←		←→?	Irises move: Induced pupils sometimes win	60:40
4	X	x	←	←** H**	Corneal reflexes move and win	93
5	X	x	x	←** J**	Eyelids move: Eyes seem to move in opposite direction.	80
6	←	←	x	←** M**	No corneal reflex. Seen veridically	87

Table 1 shows that 80% to 100% of the observers reported that the perceived directions of eye movements in Conditions 1 to 6 were the letters A D? H J M. Condition 1 is a control condition in which the pupil and iris move together, as in real life. All observers veridically reported “A.” In Condition 2, only the pupils moved and drove the perceived eye movement direction (“D”). Only in Condition 3 (only irises moving) did the observers substantially disagree. The eyes appeared to move with the irises for 60% of the observers but with the *induced* movement (Anstis & Casco 2006; Leveille & Yazdanbakhsh, 2017) of the pupils for the other 40%.

Condition 4 (stationary pupil and iris, moving corneal reflex) gave quite a convincing impression of the whole eye moving (“H”).

In Condition 5 (eyelids moving), induced movement made the stationary pupil plus iris appear to move in the opposite direction (“J”).

Condition 6 was the same as the control Condition 1 but with the corneal reflex removed. The eyes now looked spooky and dead, but the percepts were still veridical (“M”).

*Effects of contrast*. In Condition 3, the observers split between attending to the real motion of the iris and to the induced motion of the pupil. So we now moved the irises and pupils back and forth in opposite directions at 0.5 Hz in online Movie 2 and again asked the observers to report the apparent direction of the eye movements. We now systematically varied the *relative contrast* of the iris and the pupil by varying the luminance of the achromatic iris. A method of constant stimuli yielded the point of subjective equality, at which the iris and pupil were equally likely to drive the perceived direction of eye movements. For three practiced observers, these points of subjective equality occurred when the ratios of the Michelson contrasts of the pupil/iris and the sclera/iris borders were 1.02, 1.08, and 1.08. These ratios are all close to *unity*, showing that whichever had the *higher contrast*, the pupil or the iris, drove the perceived eye movement. Nothing else mattered; for instance, although the small size of the pupils might have imparted induced movement into them (Anstis & Casco, 2006; Leveille & Yazdanbakhsh, 2017), this did not change judgments of eye movement direction.

Face eccentricity affects perceived gaze direction (Todorović, 2009). Dissociating pupil and iris now introduces a second layer of eccentricity. Thus, in Figure 1(a) (iris shifted left and pupil centered), gaze appears slightly shifted in the opposite direction, that is, pupil–iris, signaling gaze-right, dominates pupil–socket that signals gaze-straight-ahead. In Figure 1(b) (pupil shifted left and iris centered), gaze appears shifted further to the left. Pupil–socket and pupil–iris relations both indicate leftward gaze, and only iris-socket relation indicates straight gaze. In Figure 1(c) (pupil and iris both shifted left), gaze appears shifted strongly left. In this nested system, the pupil is shifted left with respect to iris, and both are shifted left with respect to socket. In sum, perceived gaze direction = pupil–socket eccentricity + pupil–iris eccentricity + iris–socket eccentricity.

**Movie 1.** Click to play.

**Movie 2.** Dark iris: Iris wins. Light iris: Pupil wins.

**Figure 1.**
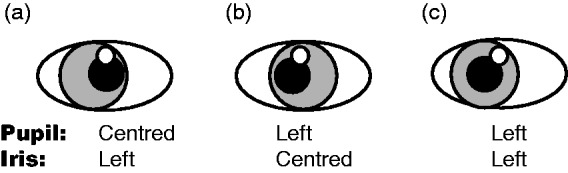
Gaze appears to be shifted (a) slightly right, (b) left, and (c) strongly left (see text).

## Conclusion

When we artificially separated the movements of pupils and irises, the direction of gaze depended upon the pupils or the corneal reflex, not the irises. When the pupils and irises artificially moved in opposite directions, the one with the higher Michelson contrast won out.
